# Short-term prediction of solar energy in Saudi Arabia using automated-design fuzzy logic systems

**DOI:** 10.1371/journal.pone.0182429

**Published:** 2017-08-14

**Authors:** Majid Almaraashi

**Affiliations:** Department of Computer Sciences, College of Computing and Information Technology, University of Jeddah, Jeddah, Saudi Arabia; Universita degli Studi della Tuscia, ITALY

## Abstract

Solar energy is considered as one of the main sources for renewable energy in the near future. However, solar energy and other renewable energy sources have a drawback related to the difficulty in predicting their availability in the near future. This problem affects optimal exploitation of solar energy, especially in connection with other resources. Therefore, reliable solar energy prediction models are essential to solar energy management and economics. This paper presents work aimed at designing reliable models to predict the global horizontal irradiance (GHI) for the next day in 8 stations in Saudi Arabia. The designed models are based on computational intelligence methods of automated-design fuzzy logic systems. The fuzzy logic systems are designed and optimized with two models using fuzzy c-means clustering (FCM) and simulated annealing (SA) algorithms. The first model uses FCM based on the subtractive clustering algorithm to automatically design the predictor fuzzy rules from data. The second model is using FCM followed by simulated annealing algorithm to enhance the prediction accuracy of the fuzzy logic system. The objective of the predictor is to accurately predict next-day global horizontal irradiance (GHI) using previous-day meteorological and solar radiation observations. The proposed models use observations of 10 variables of measured meteorological and solar radiation data to build the model. The experimentation and results of the prediction are detailed where the root mean square error of the prediction was approximately 88% for the second model tuned by simulated annealing compared to 79.75% accuracy using the first model. This results demonstrate a good modeling accuracy of the second model despite that the training and testing of the proposed models were carried out using spatially and temporally independent data.

## Introduction

Solar power is a promising resource that has the potential to deliver efficient, reliable, and easily accessible energy. However, the energy at the Earth’s surface is associated with the variability of solar radiation. In addition to the variability of solar radiation, which is related to different types of topography, weather, and geography, one of the main obstacles to solar energy adoption is the difficulty in predicting its availability. To best exploit solar energy, a reliable estimation method and prediction process is essential. Reliable knowledge of the solar resource at any location is required by solar engineers, architects, agriculturists, and hydrologists in many solar energy applications [1]. The process of solar radiation prediction is concerned with future radiation, which is characterized by a certain degree of uncertainty. This uncertainty is related to uncertainty about future weather conditions. Therefore, the prediction process, especially for short-term periods, is clearly difficult for researchers. For the sake of obtaining good predictors, many prediction and estimation models of solar radiation have been proposed in the open literature including numerical weather prediction (NWP) and artificial intelligence (AI) models (i.e., see [2] [3] [4] [5] [6] [7] [8]). Fuzzy logic systems have been applied successfully to a large number of problems in different application areas with successful modeling [9]. One of these applications is the system modeling of human knowledge and approximation of non-linear and dynamic systems. Fuzzy logic system are well known for their ability to handle uncertainty [10].

In this work, the prediction of solar energy using a computational intelligence model is carried out, to predict the solar radiation for a short-term period to help operators to better manage their solar energy systems in more economical and efficient ways. The proposed methodology is a combination of fuzzy c-means, simulated annealing and fuzzy logic systems which is applied to measured data in 8 different stations in Saudi Arabia. The rest of the paper begins with an overview of related background information in next section. Details of the methodology used are presented in Section Methodology while the results and analysis are discussed in Section Results and Discussion. Finally, the conclusions and avenues for future work are highlighted in Section Conclusion and Future Works.

## Background

The used methodology is based on an automated-design fuzzy logic system. The fuzzy logic model is designed and optimized in two stages using fuzzy c-means and simulated annealing algorithms. The objective of the predictor is to predict next-day solar radiation using meteorological and solar radiation observations. Here, the problem and its considerations as well as the methods used in the literature is reviewed.

### Short-term solar radiation prediction

The average amount of radiation from the Sun per unit area that reaches the Earth’s atmosphere for a mean solar distance is known as the solar constant, which has an approximate value of 1.360 *kW*/*m*^2^ [11]. The importance of solar radiation data has been widely observed for the design and the operation of solar energy cells and systems [12]. Therefore, information on solar radiation and its components at a given location is essential. However, the limited coverage of radiation-measuring networks raises the importance of using solar radiation prediction models. [13].

Prediction models aim to estimate the future solar radiation at a specific point on Earth. Despite its importance, future solar radiation prediction is surrounded by uncertainty. This uncertainty is related to unknown future weather conditions. Therefore, the prediction process, especially for the short term, is challenging for researchers and power operators.

### Solar energy and radiation models in Saudi Arabia

Solar projects appeared early in the Kingdom of Saudi Arabia (KSA) in the 1970s. Providing power for remote villages was the goal of Solar Village Project in the 1980s. In the 2000s, the Kingdom gave more importance to solar energy for several reasons, the growing energy demand from 35 GW in 2008 to an estimated 70 GW in 2023; power shortages during peak hours; and environmental obligations like reduction of CO2 emissions. The real hope of the Kingdom is to become a main player in solar energy as it is for oil. A recent study reveals encouraging measurement numbers regarding the amount of average daily global horizontal irradiance (GHI) in KSA ranging from 5700 *w*/*m*^2^ to 6700 *w*/*m*^2^ [14]. KSA established the King Abdullah City for Atomic and Renewable Energy (KACARE) in April 2010 to achieve the vital aim of building a substantial alternative energy capacity. Subsequently, KACARE established a project called Renewable Resource and Environmental Measurement and Monitoring [15] to obtain a map of renewable energy sources throughout Saudi Arabia using distributed measurement stations. Although, this project can meet a portion of the needs by providing vital information about solar and other renewable energy sources, short-term prediction is not yet part of the project [16].

Regarding research efforts related to solar radiation in KSA, some works have been conducted aimed at predicting solar radiation in KSA with different time scales and objectives. Hepbasli and Alsuhaibani presented a good review of the previous research on solar energy models in KSA, where models were mainly categorized into empirical (correlation) and artificial intelligence models [17].

### Fuzzy logic systems

Fuzzy logic systems are among the most well-known computational intelligence methods in AI studies. Fuzzy logic system methods have been used for modeling of a wide range of real-world problems in different application areas [18]. One of these applications is system modeling and approximation, where modeling human knowledge or approximating non-linear or dynamic systems is carried out using fuzzy logic systems. Although there is a good potential for fuzzy logic systems to model short-term solar radiation, little research has been carried out aimed at exploiting fuzzy logic systems for such a problem [19]. One important note is that the the majority of current works employ heuristic and manually configured fuzzy logic systems. However, due to the complexity associated with this problem, the automatic configuration of fuzzy logic rules becomes more appealing.

Fuzzy logic systems are rule-based systems that use the theory of fuzzy logic and fuzzy sets. Fuzzy sets theory was first proposed by Zadeh in 1965 [20]. Fuzzy logic systems have become one of the most used application of the theory. Many applications have been proposed using fuzzy logic systems to represent human knowledge in a closer way to human thinking. Unlike ordinary crisp set theory where membership is represented by two values only (0 or 1), fuzzy set theory represents any element in the set using a degree of membership of that set. Fuzzy logic systems involve the process of fuzzifying crisp input values, using the inference engine to link knowledge with fuzzy rules, and ends by defuzzifying the output fuzzy sets into normal values as outputs [9] [10]. Fuzzy logic systems can be built with a large number of different components from fuzzification operators to aggregation and defuzzification methods allowing more abilities to model different types of applications [21]. Fuzzy logic system knowledge rules can be derived from experts or using previous observations of the problem. A well-known approach to learning and tuning fuzzy logic systems is using search algorithms such as genetic algorithms and simulated annealing. Few researchers have studied the use of simulated annealing to optimize fuzzy logic systems [22]. Those who have studied this combination include [23] [24] [25] and [26]. From a methodological perspective, [25] investigated the impacts of using fuzzy c-means clustering before applying a simulated annealing search with different configurations. This research investigates the design of a high-performing predictor of solar radiation over KSA using a promising combination of fuzzy logic with fuzzy c-means and simulated annealing algorithms.

### Fuzzy c-means clustering algorithm

A cluster refers to a group of entities that have similar features. The fuzzy c-means algorithm (FCM) is a well known clustering algorithm that is based on the theory of fuzzy sets and it aims to find a number of clusters within a number of iterations using an objective function. FCM was firstly proposed by [27] and later enhanced by [28]. In fuzzy clustering, points can have different grades of memberships in different clusters rather than binary grades of memberships. FCM aims to minimize the following objective function [28]:

$$\begin{array}{c} C=\sum_{i=1}^{n}\sum_{j=1}^{m}{\left(\mu_{ij}\right)}^{f}{\left\|X_{i}-V_{j}\right\|}^{2} \end{array}$$

Where *X* = *x*_1_, *x*_2_, , , *x*_*n*_ refers to data points being clustered and *V* = *v*_1_, *v*_2_, , , *v*_*m*_ refers to cluster sets where *m* > 1. *f* is a fuzzy value which constitutes the degree of fuzziness, and it is application dependent. Each membership in fuzzy clusters must fulfill this condition:

$$\begin{array}{c} \sum_{i=1}^{n}u_{ij}=1 \end{array}$$

The number of clusters can be heuristically chosen or automatically determined using algorithms such as the subtractive clustering algorithm chosen here. In this work, FCM will be used to automatically find clusters of fuzzy membership functions by searching for the best configurations based on the known target model output from historical observations.

The subtractive clustering algorithm uses a measure of potential for each data point where this potential is reduced if new cluster center is found. It is considered as a good choice for estimating cluster centers and initial values for iterative clustering algorithms such as FCM [29]. The subtractive clustering algorithm determines the potential of each data point *x*_*i*_ at first by the following formula [29]:

$$\begin{array}{c} P_{i}=\sum_{i=1}^{n}e^{-\alpha ∥x_{i}-x_{j}∥} \end{array}$$

Where

$$\begin{array}{c} \alpha =\frac{4}{r_{a}^{2}} \end{array}$$

and *r*_*a*_ is a positive constant called cluster radius that defines neighborhood.

### Simulated annealingalgorithm

The simulated annealing algorithm (SA) is a simple and general optimization algorithm for finding global minima introduced in [30]. It has been used widely to search for optimal or nearly optimal solutions in a wide range of optimization problems. SA uses the Metropolis algorithm to imitate metal annealing in metallurgy, where heating and controlled cooling of materials are used to reshape metals. It has been used for a large number of problems to search for optimal or nearly optimal solutions. In this work, SA will be used as a learning algorithm to automatically design fuzzy logic systems by searching for their best configurations. Traditionally, experts have been able to provide efficient rules for designing simple fuzzy logic systems with few inputs. However, this is no longer always the case due to growing complexity and increasing uncertainty, which makes the rule base and membership functions difficult to acquire. In such cases, automated learning methods such as genetic algorithms or simulated annealing have to be used to optimize fuzzy logic systems outputs. In general, SA can find good solutions for a wide range of problems, though often at the cost of increased running times [31]. In addition, the use of simulated annealing does not require the existence of some mathematical properties such as differentiability in the problem when optimizing fuzzy logic systems [19]. This feature adds more flexibility by allowing the use of all fuzzy structure components. SA works by starting to accept improving states while gradually reducing the probability of accepting bad states. This probability is a function of a control parameter called temperature. An adequate temperature scheduling is important to optimize the search. The choice of good parameters is important for the success of SA to avoid getting stuck in local minimas and to avoid unneeded, excessive searches. One of the ways to reduce the optimization time and computations is to initialize the configuration of fuzzy logic systems using a clustering algorithm such as fuzzy c-means followed by more tuning of the simulated annealing.

## Methodology

**Fig 1:**
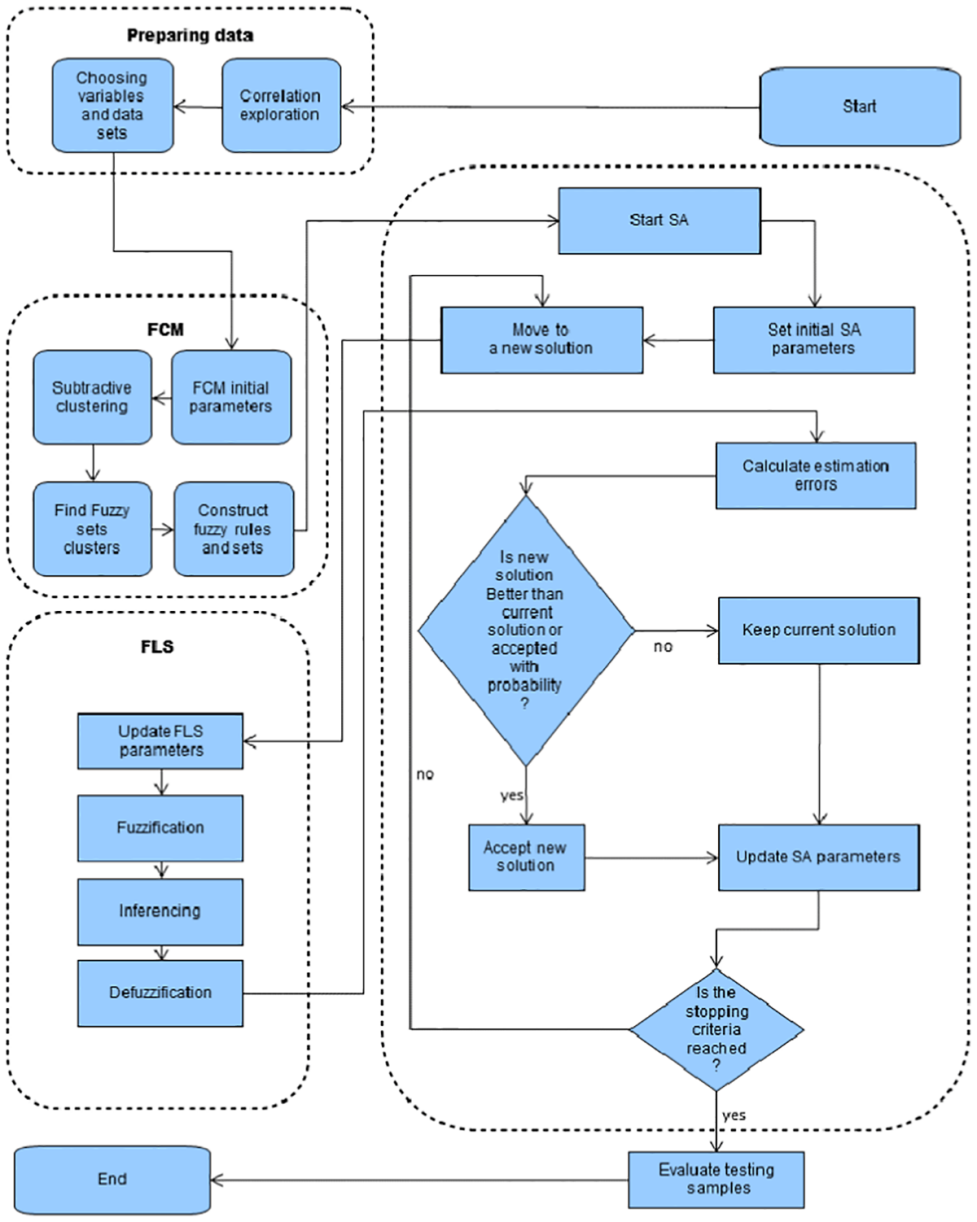
A flowchart of the used methodology to predict daily solar radiation.

The experiment can be divided into four steps: preparing data, constructing the initial fuzzy logic system using FCM, learning the fuzzy logic system parameters using SA and testing new data set using the best fuzzy logic model found. The experiment is illustrated further by the flowchart in Fig 1.

### Preparing data sets

In order to predict solar radiation, the system will use historical observed data. From the works reported in the literature, a notable variations in the selection of the input variables to prediction models has been reported [32]. The proposed method uses available weather and solar radiation data to build the model that predicts next-day Global Horizontal Irradiance (ND-GHI). All ten variables used as inputs are listed in Table 1 including temperature, humidity, wind speed and some solar radiation data. The weather and solar radiation variables used are considered as inputs to the predictor system while solar radiation of the next-day is the target output. Each of these input parameters will be modeled using input fuzzy sets. The model output is the predicted global horizontal irradiance (GHI) for the next day, which is modeled using an output fuzzy set. Historical data will be divided into two separate groups: training and testing samples used in the two processes. The training process aims to optimize the parameters of the antecedent and consequent parts of the fuzzy logic system rules.

**Table 1:** The model inputs and output in this experiment with their correlation coefficient and p-values.

Input Variables	Abbrev.	Correlation coefficient	P-Values
Month	M	-0.13	3.266e-08
Air Temperature (Degrees C)	T	0.67	4.796e-224
Avg Wind Direct at 3^m^ (Deg from North)	WD	0.09	8.427e-05
Avg Wind Speed at 3^m^ (m/s)	WS	0.13	3.989e-08
Diffuse Horizontal Irradiance (DHI) (*Wh*/*m*^2^)	DHI	0.34	9.892e-48
Direct Normal Irradiance (DNI) (*Wh*/*m*^2^)	DNI	0.41	1.130e-68
Global Horizontal Irradiance (GHI) of current day (*Wh*/*m*^2^)	GHI	0.89	0
Peak Wind Speed at 3m (m/s)	PWS	0.18	1.386e-13
Relative Humidity (Percent)	RH	-0.59	1.821e-160
Station Pressure (mB (hPa equivalent))	P	-0.13	5.504e-08
Next-day GHI (*Wh*/*m*^2^) (model output)	ND-GHI	-	-

The data used is for 8 stations in 8 cities that have been installed and are monitored by KACARE as part of the Renewable Resource Monitoring and Mapping (RRMM) Program [15]. A sample of the used data is shown in Table 2 that corresponds to Riyadh station. Before constructing the fuzzy logic system predictor, a correlation test is carried out to check whether all these variables have a minimum correlation with the output. The correlation coefficients and p-values are calculated as shown in Table 1, which shows correlation coefficients between −0.13 as a minimum coefficient and 0.89 as the most correlated variable while all p-values are very close to zero indicating a good correlation. Therefore, we choose use all the ten variables in this study. In addition, we observed that two other associated variables were not recorded in the ground stations in this period due to technical issues, which include sky cover and visibility parameters as well as the uncertainty values associated with all measured data. A future work will investigate adding more related variables from other sources that might enhance the prediction performance.

**Table 2:** A sample of the data used in this experimentation for Riyadh station.

M	T	WD	WS	DHI	DNI	GHI	PWS	RH	P	ND-GHI
6	34.9	340	4.5	3552	5389	8121	14.7	8	934.4	7098
6	34.5	341	5.6	4076	4006	7098	17.3	7.9	934.3	7378
6	34.6	337	5.3	4842	2894	7378	16	9.5	933.6	7864
7	38	314	2.8	1955	8217	8151	11.7	8.1	927.7	7664
7	37.2	344	3.4	2352	7190	8024	13.9	10.3	928.8	7894
7	36.7	339	4.1	2845	6228	7894	17.3	8.7	928.2	6921
8	36.4	316	2.6	2137	7726	7961	10.1	9.3	928.2	7798
8	35.9	315	3.3	2347	7251	7798	12.5	9.1	928.8	7523
8	35.6	315	3.9	3046	5581	7523	13.1	9.3	928.7	7104
9	38.6	131	2.3	3337	3657	6224	8.3	14.9	933.2	6175
9	39.2	86	2.5	2598	4499	6175	9.9	12.3	933	6362
9	39.2	285	2.1	2416	5107	6362	7.7	9.4	931.5	7010
10	32	62	3.1	1409	7888	6586	9.3	13.1	934.5	6416
10	31.5	107	2.4	1647	7241	6416	7.2	18	936.2	6105
10	32.5	89	2	1793	6540	6105	8	14.1	937.2	6337
11	26.7	123	2.6	1176	7005	5286	8.3	28.3	939.3	5019
11	27.5	145	3.6	1705	5548	5019	10.4	27.1	937.7	5114
11	28.8	187	2.7	1522	6025	5114	9.6	25.6	937.5	3596
12	20.8	327	2.3	845	7502	4705	8.8	41.8	945.7	4641
12	19.5	332	2	941	7040	4641	6.4	49.9	945.5	4747
12	21.9	240	2	776	7768	4747	6.7	35	941.1	4734
1	15.9	9	3.5	2311	2589	3718	9.9	41.5	941	3972
1	15.1	56	3.1	2030	3648	3972	9.3	47.1	943.8	2863
1	14.2	111	2.5	2256	1127	2863	6.9	62.1	945.1	4251
2	17.5	96	2.3	2274	3676	4519	6.9	48.7	940.1	4746
2	17	125	3.3	2057	4422	4746	10.4	50.2	937.1	5051
2	18.3	144	3.4	1423	6170	5051	10.4	54.6	934	5025
3	21.1	53	3.6	1447	7773	6365	9.6	35.5	940.4	5802
3	21.8	113	2.1	2181	5404	5802	7.2	29.1	940.8	4573
3	24.1	124	2.1	3350	1680	4573	7.2	25.8	938.6	3873
4	23.1	118	3.7	3964	3430	6685	9.9	22.2	935.3	4741
4	24.8	147	3.7	3643	1330	4741	15.7	39.3	931.2	6859
4	20.1	354	4.1	3697	4028	6859	11.5	45.7	935.1	7099
5	31.3	155	2.8	4079	3095	6791	10.4	29.7	933.4	6122
5	33.3	198	2.6	4302	2334	6122	9.1	20.2	932.8	6703
5	30.9	28	4.2	4115	3380	6703	16	23.7	935	4653

**Fig 2:**
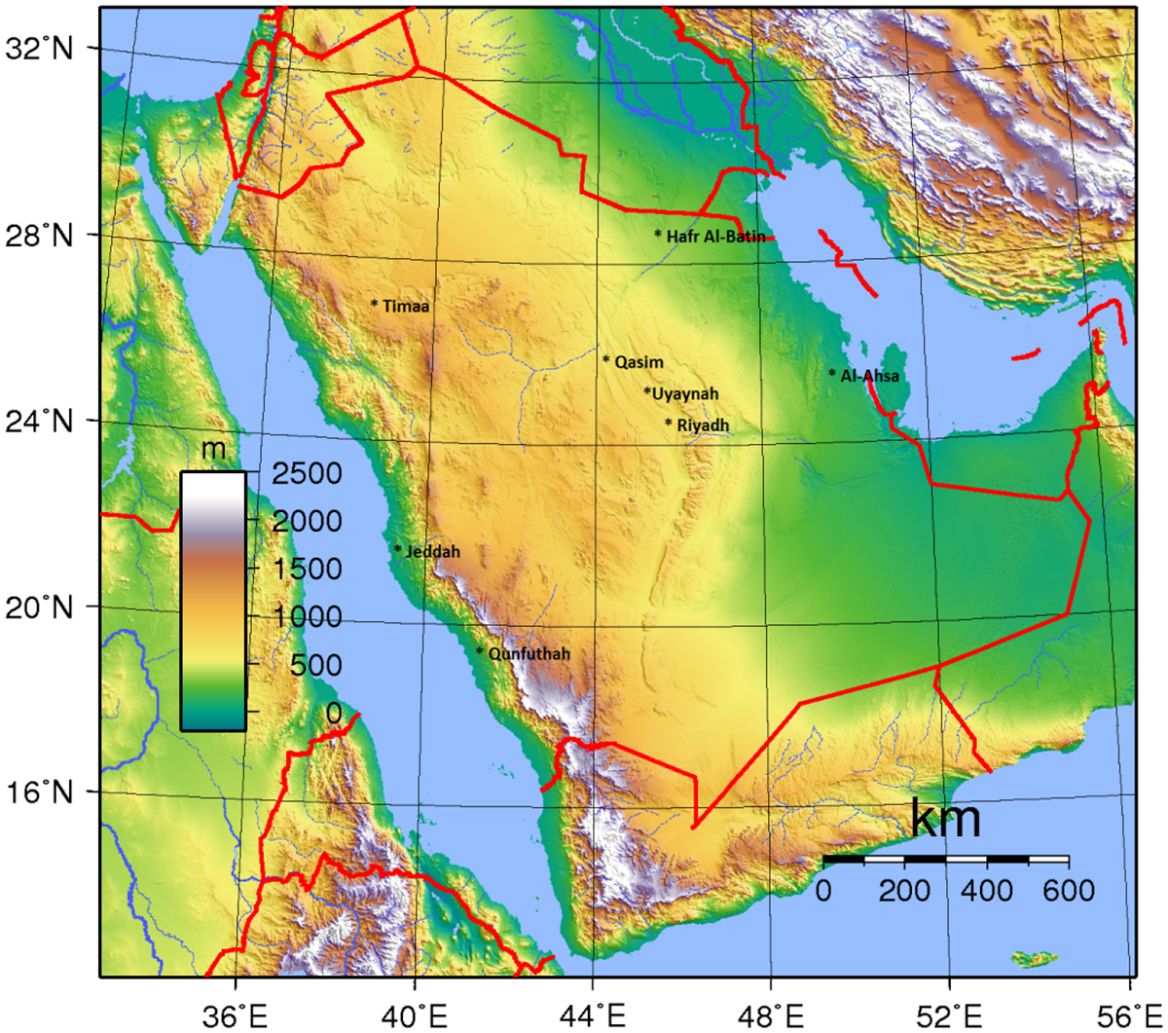
The locations of used stations in this study in Saudi Arabia (amended from [33]).

The data includes daily observations for 582 days from mid-2013 to the end of 2014 with missing values. The training process uses data sets for five different locations to build the model as shown in Table 3. Other data sets for three different locations are used to test the model. Namely, the training process uses historical data for Riyadh, Jeddah, Qasim, Timaa and Al-Ahsa cities for one year period from 5th June 2013 to 4th June 2014 (365 days). Then, the testing process is applied on data for Hafr Al-Batin, Al-Qunfudhah and Al-Uyaynah cities for the rest of 2014 till 30th December (209 days). Fig 2 shows a map including these locations. The previous one-day observations of all variables prior to the predicted day are used for next-day prediction in training and testing stages. Regarding the cleaning of the used data, there are 123 missing records within the training data in which 1702 records are used instead of 1825.

**Table 3:** The details of the stations selected for the experimentation.

Purpose	Station name	Latitude	Longitude	Elevation (m)	Full Name
Training	Riyadh	24.70814	46.67896	668	K.A.CARE Building Olaya St
Training	Jeddah	21.49604	39.24492	75	KingAbdulaziz University Main Campus
Training	Al-Ahsa	25.34616	49.5956	170	King Faisal University
Training	Qassim	26.34668	43.76645	688	Qassim University
Training	Timaa	27.6173	38.5252	844	Timaa Technical Institute
Testing	Hafar-Al-Batin	28.33202	45.95708	383	Hafar AlBatin Technical College
Testing	Al-Qunfudhah	19.15197	41.08111	20	Al Qunfudhah Technical Institute
Testing	Al-Uyaynah	24.90689	46.39721	779	Al-Uyaynah Research Station

### Constructing the initial fuzzy logic system using fuzzy c-means

The idea behind using a fuzzy clustering algorithm is to find a number of clusters that will be represented as fuzzy rules. Therefore, the number of clusters determines the number of rules and membership functions in the constructed fuzzy logic system. In this work, the number of fuzzy logic rules and membership functions are determined by the number of clusters chosen. To get a suitable number of clusters automatically, the subtractive clustering algorithm was used for determining the number of clusters automatically with a cluster influence range (radius) equals to 0.5. The minimum and maximum values of the data set are used as the minimum and maximum normalization bounds for each data dimension. Therefore, FCM will start from the number and centers of clusters found by the subtractive clustering algorithm.

Thereafter, fuzzy c-means finds the number of clusters (rules) where each variable is divided into a number of membership functions equal to the number of rules. The fuzzy logic system has ten inputs and one output while choosing the Mamdani-type fuzzy logic system and Gaussian membership function as the shape of all fuzzy sets in both the input and the output fuzzy sets. The Gaussian membership function has two parameters, which are the mean *m* (center) and the standard deviation *σ*, and it is determined by the following formula:

$$\begin{array}{c} \tilde{f}\left(x\right)=\exp^{-{\left(\frac{x-m}{2\sigma }\right)}^{2}} \end{array}$$ (1)

All the centers and standard deviations of all fuzzy sets are initially set by using the fuzzy c-means algorithm, as described above. The use of the fuzzy c-means algorithm resulted in founding 30 clusters that were converted to 30 rules with 30 * 11 = 330 fuzzy sets. Samples of the fuzzy logic system rules constructed by fuzzy c-means are shown in Table 4. Then, the fuzzy logic system was constructed by applying the fuzzification, implication and defuzzification processes. The fuzzification and the implication processes are both based on the minimum operation. The aggregation process is based on the maximum operation while the centroid process is chosen for the defuzzification process. An example of the designed fuzzy logic system rules using FCM is shown in Table 4. After constructing the fuzzy logic systems, the error of estimating the training and testing samples using the constructed fuzzy logic system is evaluated.

**Table 4:** Samples of the fuzzy logic system rules constructed by fuzzy c-means. Abbreviations “in” and “out” refer to input and output variables respectively while “mf” refers to membership functions.

1	If (in1 is in1mf1)	(in2 is in2mf1)	.….	(in10 is in10mf1)	Then (SR is out1mf1)
2	If (in1 is in1mf2)	(in2 is in2mf2)	.….	(in10 is in10mf2)	Then (SR is out1mf2)
3	If (in1 is in1mf3)	(in2 is in2mf3)	.….	(in10 is in10mf3)	Then (SR is out1mf3)
4	If (in1 is in1mf4)	(in2 is in2mf4)	.….	(in10 is in10mf4)	Then (SR is out1mf4)
5	If (in1 is in1mf5)	(in2 is in2mf5)	.….	(in10 is in10mf5)	Then (SR is out1mf5)
6	If (in1 is in1mf6)	(in2 is in2mf6)	.….	(in10 is in10mf6)	Then (SR is out1mf6)
.…….	.……….	.……….	…………	…………	.……….
.…….	.……….	.……….	…………	…………	.……….
30	If (in1 is in1mf30)	(in2 is in2mf30)	.….	(in10 is in10mf30)	Then (SR is out1mf30)

### Learning the fuzzy logic system parameters using simulated annealing

The second optimization process of membership functions designed by FCM is done using SA algorithm that searches for the best combination of these parameters by trying to generate new values for the parameters each time. Then, it will evaluate the cost of the new state, which is measured by an error function, the root mean square error (RMSE), which represents the cost function that is to be minimized. The RMSE is chosen because it is measured in the same scale as the data. The RMSE as the objective function is defined as follows:

$$\begin{array}{c} RMSE=\sqrt{\frac{1}{n}\sum_{i}^{n}{\left(f\left(x\right)-\hat{f}\left(x\right)\right)}^{2}} \end{array}$$ (2)

where *n* is the number of data samples in the observed data set, *f*(*x*) is the output of the trained fuzzy logic system, and $\hat{f}\left(x\right)$ is the target output that the trained system aims to approach. The total number of optimized parameters (centers and standard deviations) in the model is 30 * 11 + 30 * 11 = 660. The objective is to find the set of these parameters that best minimizes the prediction error of the training samples.

The SA algorithm is initialized with a temperature set to 20 and a cooling schedule based on Boltzman annealing by updating the current temperature in each iteration based on the initial temperature *T*_0_ and the current iteration number *k*_*i*_ using the following formula:

$$\begin{array}{c} T_{i}=T_{0}/\ln\left(k_{i}\right) \end{array}$$

The temperature is re-annealed after accepting a certain number of new states equals to 1760 iterations to allow more diverse search. The neighboring solutions for a current solution are chosen randomly by generating new solutions based on the current solution and the current temperature using multivariate normal distribution with step sizes equal to the square root of the temperature and with a uniformly random direction. The search ends after a number of iterations proportional to the number of parameters, which is 660 * 25 = 16500 iterations. The experimentation is repeated 50 times, and the results of all runs are recorded.

### Testing the proposed model using new data set

After optimizing the fuzzy logic system using training samples, testing samples are evaluated by examining the outputs of the 209 data samples for the other three stations. The objective of this process is to test the generality of the found model using unseen data referring to future cases but using the same inputs used with training samples. To certify the generality of the model, the training and testing of the proposed model were carried out using spatially and temporally independent data. The choice of training and testing locations were based on the availability of data as well as choosing locations with different topographies. The average, maximum, minimum, and standard deviations of the training and testing RMSEs have been calculated.

## Results and discussion

**Fig 3:**
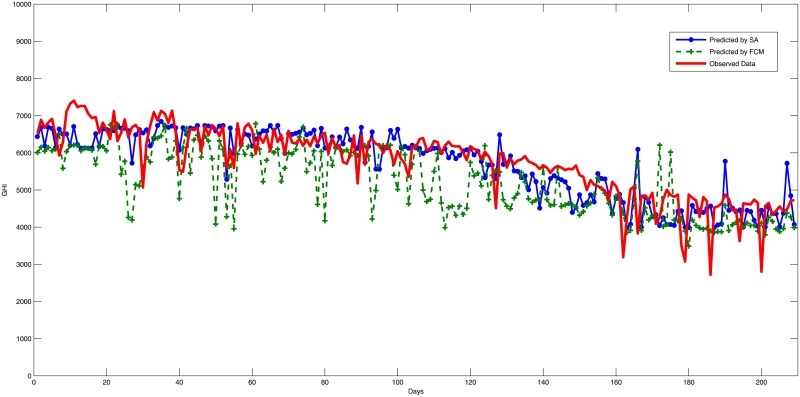
Estimation errors (RMSE) during testing for Qunfuthah city using fuzzy c-means and simulated annealing algorithms.

**Fig 4:**
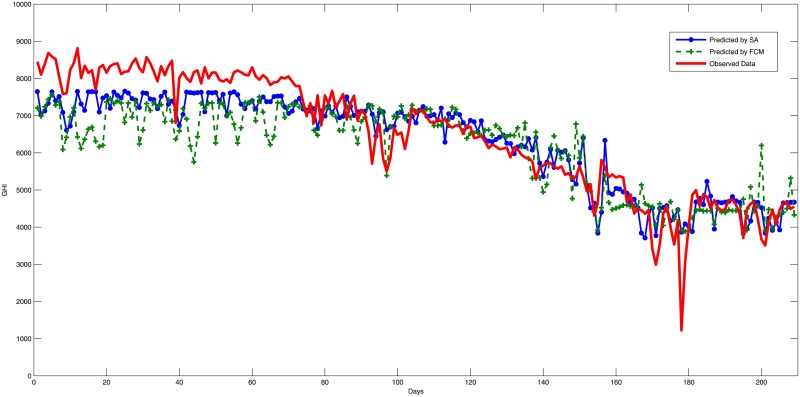
Estimation errors (RMSE) during testing for Uyaynah city using fuzzy c-means and simulated annealing algorithms.

**Fig 5:**
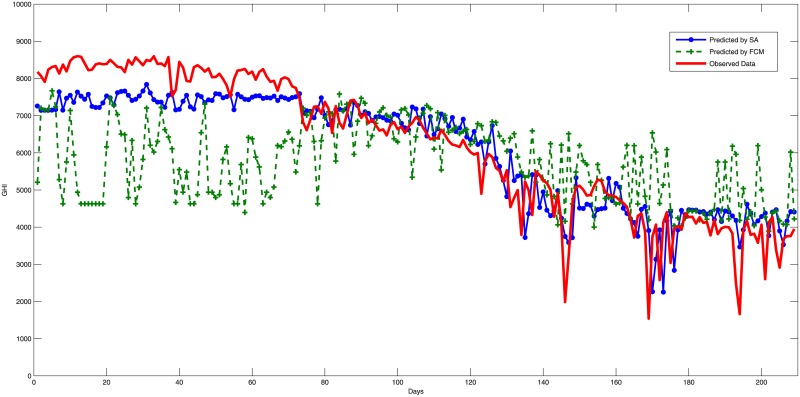
Estimation errors (RMSE) during testing for Hafr Al-Batin city using fuzzy c-means and simulated annealing algorithms.

The experiment has been carried out 50 times using Matlab. Summaries that describe the data and Estimation error results are shown in Tables 5 and 6 while the estimation performance for the testing phase are shown in Figs 3, 4 and 5. The main results and discussions are as follows:

- The training results revealed that SA has outperformed FCM as it reduces the training errors from an average of 945.07 to 769.2 with an extra improvement about 0.3 of the average training data of 6014.6.
- The construction of a fuzzy logic system by FCM clustering obtained an average RMSE of 1247.32 for the three testing samples with an average minimum RMSE of 1173.35, which represent 20.25% and 19% from the average of the measured values, respectively. Thus, FCM has achieved an average estimation accuracy of 79.75%. Figs 3, 4 and 5 show prediction results of the used models compared to the real data, in which the FCM clustering fails to capture some of the data trends and outliers. However, FCM constructed a good solution for SA to start the search with rather than starting the search from scratch.
- The fine tuning of the fuzzy logic system constructed by FCM and SA has achieved an average RMSE of 725.27 and a minimum RMSE of 641.02, which represent 11.78% and 10.41% compared to the average of the measured values, respectively. Therefore, the combination of the two algorithms has achieved an average good estimation accuracy of 88.22%. In addition, the figures show the good performance of the SA tuning, which achieved results that captured the main trends and outliers of the data noticeably better than the FCM. To get an idea of how good these models, Table 7 shows some results from the literature and reveals that the model of fuzzy logic systems optimized by FCM and SA achieved a good performance compared to other models. The overall results of the model agree with the performance range reported by other researchers despite that we choose to use different training and testing locations with different temporal properties which is not applied normally by other works. This is an important indicator of the generalization ability of the model. On the other hand, the FCM has not achieved a good configuration of fuzzy logic systems due to the lack of generalization in the clustering process of FCM.
- The good generality of the proposed model achieved in this study should allow using the model to predict solar radiation for new locations in Saudi Arabia with a good reliability and expectation of the model performance.
- The whole experimentation has taken an average 980 seconds for each run while the FCM clustering has been achieved in an average of 3 seconds. The SA has carried out the search within the other 977 seconds which equals to about 16 minutes. However, the extra performance of SA over FCM can justify the extra computational time. The total time reported should be acceptable by solar operators to plan next-day operations allowing the model to be a good option in practice.

**Table 5:** Measured GHI data summary.

Data	*SR*_*Average*_	*SR*_*Min*_	*SR*_*Max*_	*SR*_*STD*_
Training Data	6014.6	391	8864	1517.9
Testing Data (Hafr Al-Batin)	6221.5	1538	8599	1833.4
Testing Data (Qunfuthah)	5771.6	2716	7403	921
Testing Data (Uyaynah)	6482.6	1220	8812	1566.9
Testing Data (All cities)	6158.5	1824.66	8271.33	1440.43

**Table 6:** Experimental Results using fuzzy c-means and simulated annealing in training and testing phases.

Runs	Data set	*RMSE*_*Average*_	*RMSE*_*Min*_	*RMSE*_*Max*_	*RMSE*_*STD*_
FLS by FCM	Training	945.07	929.87	973.87	7.86
FLS by SA	Training	769.2	738.6	796.5	12.26
FLS by FCM	Qunfuthah (Testing)	1038.6	892.65	1307	125.47
FLS by FCM	Uyaynah (Testing)	912.47	877.61	952.95	16.09
FLS by FCM	Hafr Al-Batin (Testing)	1790.9	1749.8	1841.9	28.07
FLS by SA	Qunfuthah (Testing)	693.36	547.09	928.76	82.34
FLS by SA	Uyaynah (Testing)	698.68	647.1	767.07	27.33
FLS by SA	Hafr Al-Batin (Testing)	783.77	728.89	875.53	34.01
FLS by FCM	All cities (Testing)	1247.32	1173.35	1367.28	56.54
FLS by SA	All cities (Testing)	725.27	641.02	857.12	47.89

**Table 7:** Comparison of proposed methods results with some other methods from the literature.

Runs	*RMSE*_*Average*_(%)
Neural Networks [34]	6.002%
Bayesian Neural Networks (different settings) [34]	7.5% − 15%
Different empirical models [34]	7.49% − 59.23%
multi-layer perceptron Neural Networks [35]	5% − 7.5%
MBE Liner model [5]	9.32%
Nonlinear model [5]	8.73%
Fuzzy logic model [5]	8.80%
Neural Networks model [5]	7.37%
Neural Networks model (different cities) [4]	9.1% − 20.5%
**FCM+FLS**	20.25%
**FCM+SA+FLS**	11.78%

In order to enhance the modeling performance, one important feature to note in future work is that the selection of used variables among available variables and the selection of optimal time intervals for each variable might add more modeling capabilities. As mentioned in subsection Preparing Data Sets, this is another research question that is currently under investigation, and it should further enhance -in theory- the accuracy of the model. In other words, taking into account that the variables used in this study do not cover the sky cover and visibility parameters due to data availability, a future work might investigate the addition of other important variables and uncertainty measures from other sources other than ground stations and the effects of the added variables on prediction performance as well as the reduction of the number of features (variables).

## Conclusion and future works

In this article, two models based on fuzzy c-means clustering (FCM) and simulated annealing (SA) are presented and applied to predict daily solar radiation through fuzzy logic systems. The first model uses FCM based on the subtractive clustering algorithm to find a number of clusters that will be represented as the predictor fuzzy rules. The second model is using FCM followed by simulated annealing algorithm to enhance the prediction accuracy of the fuzzy logic system. The prediction of solar radiation is applied to measured data in 8 stations in Saudi Arabia. The fuzzy logic models are designed and optimized using FCM and SA, in which fuzzy rules are automatically generated from data. The first model achieved 79.75% accuracy using the FCM algorithm. Further tuning of the first model using simulated annealing has increased the accuracy to 88.22%, which is in good agreement with the real data despite that we choose to use different training and testing locations with diverse spatial and temporal properties. Future work will investigate adding more variables from other resources to discover potential relations to select the most related variables for such prediction. Another future work will exploit the uncertainty values that are provided for some of the measured data to enhance the prediction accuracy.
